# β-Arrestin 2 and ERK1/2 Are Important Mediators Engaged in Close Cooperation between TRPV1 and µ-Opioid Receptors in the Plasma Membrane

**DOI:** 10.3390/ijms21134626

**Published:** 2020-06-29

**Authors:** Barbora Melkes, Vendula Markova, Lucie Hejnova, Jiri Novotny

**Affiliations:** Department of Physiology, Faculty of Science, Charles University, 128 00 Prague, Czech Republic; barbora.melkes@natur.cuni.cz (B.M.); vendula.markova@natur.cuni.cz (V.M.); lucie.hejnova@natur.cuni.cz (L.H.)

**Keywords:** μ-opioid receptor, TRPV1, β-arrestin 2, ERK1/2, biased signaling, receptor lateral mobility

## Abstract

The interactions between TRPV1 and µ-opioid receptors (MOR) have recently attracted much attention because these two receptors play important roles in pain pathways and can apparently modulate each other’s functioning. However, the knowledge about signaling interactions and crosstalk between these two receptors is still limited. In this study, we investigated the mutual interactions between MOR and TRPV1 shortly after their activation in HEK293 cells expressing these two receptors. After activation of one receptor we observed significant changes in the other receptor’s lateral mobility and vice versa. However, the changes in receptor movement within the plasma membrane were not connected with activation of the other receptor. We also observed that plasma membrane β-arrestin 2 levels were altered after treatment with agonists of both these receptors. Knockdown of β-arrestin 2 blocked all changes in the lateral mobility of both receptors. Furthermore, we found that β-arrestin 2 can play an important role in modulating the effectiveness of ERK1/2 phosphorylation after activation of MOR in the presence of TRPV1. These data suggest that β-arrestin 2 and ERK1/2 are important mediators between these two receptors and their signaling pathways. Collectively, MOR and TRPV1 can mutually affect each other’s behavior and β-arrestin 2 apparently plays a key role in the bidirectional crosstalk between these two receptors in the plasma membrane.

## 1. Introduction

Large numbers of studies conducted over the previous decades have increased knowledge of pain mechanisms at both the cellular and molecular level. Several membrane-bound receptors and ion channels have been found to play a key role in the transmission or attenuation of painful stimuli. Among them, the µ-opioid receptor (MOR) has attracted great attention as the primary molecular target of opioid drugs, a group of most effective analgesics [1,2]. MOR belongs to the family of G protein-coupled receptors (GPCRs). MOR is linked to the inhibitory G proteins, whose major signaling pathway leads to inhibition of adenylyl cyclase or modulation of mitogen activated protein kinases (MAPKs). The properties of these receptors and their signaling systems have been extensively studied not only in connection with nociception but also with a potential risk of tolerance and dependence associated with long-term use or abuse of opioids [3,4,5]. Interestingly, it has been observed that MOR-initiated signaling can be modulated by other GPCRs or some ion channels. In the case of antinociception, communication between the MOR and TRPV1 (transient receptor potential vanilloid 1) receptors appears particularly noteworthy [6,7].

TRPV1 receptors, which play a central role in thermal nociception and inflammation-induced hyperalgesia, belong to the large superfamily of TRP ion channels [8]. These are ligand-driven, non-selective cation channels that are present at low levels in different types of cells and tissues but are primarily expressed in the posterior spinal neurons and trigeminal ganglia [9]. TRPV1 receptors are considered to be important molecular integrators of nociceptive stimuli because they respond not only to elevated temperature but also to reduced pH, vanilloids (e.g., capsaicin), and other pain-inducing substances (e.g., arachidonic acid metabolites). Furthermore, these receptors serve as the final target structure of intracellular signaling pathways triggered by inflammatory mediators, thereby potentiating their activity and contributing to elevated hyperexcitability and nociception [10]. Thus, the function of TRPV1 receptors can be regulated through their direct activation by “external” pain stimuli or through “intrinsic” sensitization induced by intracellular signaling cascades that are linked to GPCRs or receptor tyrosine kinases (RTKs). The sensitization of TRPV1 receptors is based on their post-translational modifications, such as protein kinase A (PKA)- and protein kinase C (PKC)-mediated phosphorylation [11].

Previous studies have demonstrated natural co-expression of MOR and TRPV1 receptors in different regions of the central nervous system and pointed to possible functional interactions between these receptors [7,12]. In the recent past, Bao et al. [13] have noticed that MOR and TRPV1 receptor agonists may have conflicting effects on antinociception, tolerance, and dependence. It has been repeatedly observed that administration of morphine can induce antinociceptive effects by modulating the activity of TRPV1 receptors. As indicated above, TRPV1 receptors can be activated in a variety of ways. However, the potential specific effects of MOR agonists on TRPV1 receptors and vice versa have not yet been fully explored.

**Figure 1:**
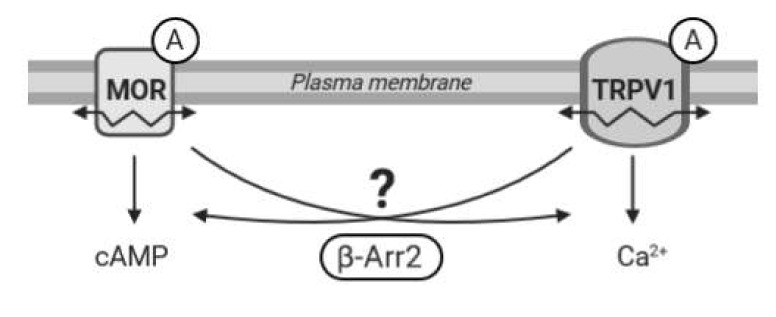
Working hypothesis. The diagram illustrates a presumed crosstalk between the µ-opioid receptor (MOR) and TRPV1 following stimulation by agonists (A). β-Arrestin 2 (β-Arr2) plays an important role in signal transduction pathways initiated by MOR and TRPV1. We hypothesize that β-arrestin 2 may also participate in mutual communication between these two receptors. We speculate that activation of one receptor may affect functioning and mobility (↭) in the plasma membrane of the other receptor and that β-arrestin 2 could mediate these interactions.

Recent evidence suggests that functional communication between MOR and TRPV1 receptors is bilateral and highly dependent on specific conditions; the mechanism and duration of activation of these receptors seem particularly important. However, many intriguing questions regarding MOR–TRPV1 interactions, crosstalk between their signaling pathways, and respective feedback loops still remain unresolved. Therefore, in the present study we set out to investigate behavior and functional properties of these receptors and their signaling pathways in defined in vitro conditions and examine the role of key components of both these important cellular signaling systems in their mutual communication (Figure 1).

## 2. Results

### 2.1. Transient Transfection of HMY-1 Cells with TRPV1–CFP

**Figure 2:**
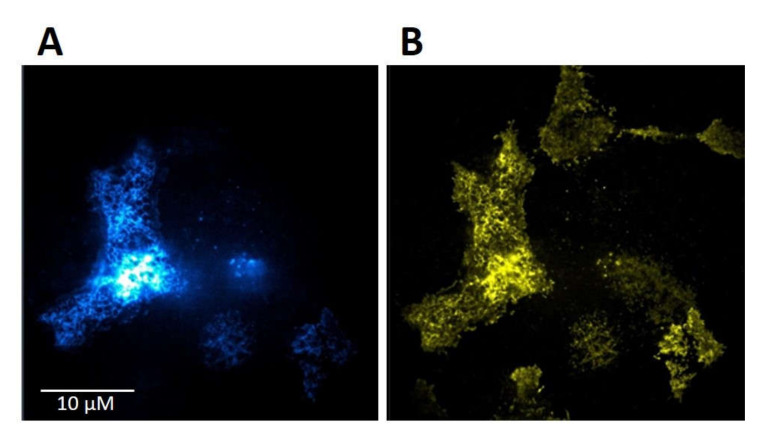
Expression of TRPV1 and MOR in HMY-1/TRPV1 cells. HMY-1 cells were transiently transfected with a construct expressing TRPV1–CFP as described in Section 4. Representative images of a group of HMY-1/TRPV1 cells (focus on the bottom cell membrane) demonstrate the distribution of the fluorescence signal corresponding to TRPV1–CFP (**A**) and MOR–YFP (**B**) in the plasma membrane. Both these photographs were taken using the TIRF mode of super-resolution microscope Zeiss Elyra.

HMY-1 cells stably expressing MOR–YFP fusion construct [14] were transiently transfected with TRPV1–CFP fusion construct as described in Section 4. The transfection efficiency of TRPV1–CFP into HMY-1 cells was >60%. The expression and plasma membrane localization of TRPV1 and MOR is shown in Figure 2A,B, respectively. This figure displays representative TIRF images of the bottom membrane of HMY-1/TRPV1 cells taken by a super-resolution microscope (Zeiss Elyra).

### 2.2. Activation of TRPV1 Affects MOR Mobility at the Cell Surface

**Figure 3:**
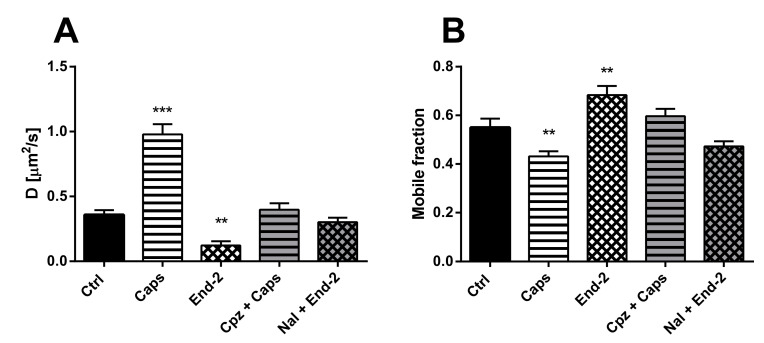
Effect of different ligands on the lateral mobility of MOR in the plasma membrane of HMY-1/TRPV1 cells. The diffusion coefficients (**A**) and the mobile fractions (**B**) of MOR were obtained from fluorescent recovery after photobleaching (FRAP) measurements. The cells plated in a glass bottom chamber were treated with capsaicin (Caps, 0.5 µM) or endomorphin-2 (End-2, 1 µM) for 5 min before measurements. In some cases, the cells were incubated in the presence of the TRPV antagonist capsazepine (Cpz) or the MOR antagonist naloxone (Nal) (both 10 µM) for 10 min prior to addition of the agonists. FRAP experiments were performed on the bottom cell membrane using a Zeiss LSM 880 confocal microscope. The data were collected from three independent experiments, at least 50 cells in each group. Results are expressed as means ± S.E.M. Asterisks denote significant differences between control (Ctrl) and different drug treatment groups (** *p* < 0.01, *** *p* < 0.001 compared to corresponding control).

First, we measured the diffusion coefficients of MOR under different experimental conditions. HMY-1 cells were seeded in a glass bottom chamber, transiently transfected with TRPV1–CFP construct and after 24-h incubation ligands of interest were added. In the case of the MOR agonist endomorphin-2, we observed changes similar to those seen in HMY-1 cells without TRPV1–CFP [14], i.e., a decrease in the rate of receptor diffusion (Figure 3A). On the other hand, the diffusion coefficient of MOR increased at least twofold after treatment of the cells with the TRPV1 agonist capsaicin (control, D = 0.358 ± 0.036 µm^2^/s; capsaicin, D = 0.978 ± 0.077 µm^2^/s). To check the specific effect of agonists, some cells were treated with antagonist before adding the agonists. Pretreatment of the cells with naloxone or capsazepine completely blocked the effects of the MOR agonist endomorphin-2 or TRPV1 agonist capsaicin, respectively.

Next, we determined the proportion of mobile and immobile receptors in the plasma membrane after activation of both MOR and TRPV1 with their cognate agonists. Whereas the mobile fraction of MOR significantly increased after treatment of HMY-1/TRPV1 cells with endomorphin-2, this fraction markedly decreased in the presence of capsaicin (Figure 3B). The effects of either agonist were prevented when the respective antagonists (naloxone and capsazepine) were added to cell culture media prior to each agonist.

### 2.3. Activation of MOR Affects TRPV1 Mobility at the Cell Surface

**Figure 4:**
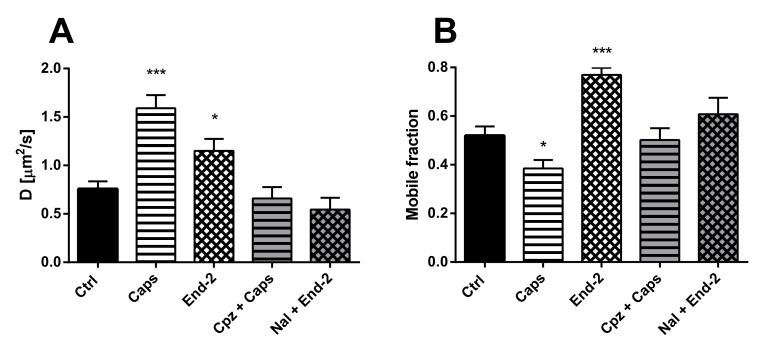
Effect of different ligands on the lateral mobility of TRPV1 in the plasma membrane of HMY-1/TRPV1 cells. The diffusion coefficients (**A**) and the mobile fractions (**B**) of MOR were obtained from FRAP measurements. The cells plated in a glass bottom chamber were treated with capsaicin (Caps, 0.5 µM) or endomorphin-2 (End-2, 1 µM) for 5 min before measurements. In some cases, the cells were incubated in the presence of the TRPV antagonist capsazepine (Cpz) or the MOR antagonist naloxone (both 10 µM) for 10 min prior to addition of the agonists. FRAP experiments were performed on the bottom cell membrane using a Zeiss LSM 880 confocal microscope. The data were collected from three independent experiments, at least 50 cells in each group. Results are expressed as means ± S.E.M. Asterisks denote significant differences between control (Ctrl) and different drug treatment groups (* *p* < 0.05, *** *p* < 0.001 compared to corresponding control).

The diffusion of unactivated TRPV1 (D = 0.75 ± 0.07 µm^2^/s) was higher than the diffusion of unactivated MOR (D = 0.36 ± 0.03 µm^2^/s) in cells expressing both these receptors. After activation of TRPV1 with capsaicin, the diffusion coefficient of this receptor increased more than two times (D = 1.590 ± 0.136 µm^2^/s), compared to the control vehicle-treated cells (D = 0.73 ± 0.09 µm^2^/s). Moreover, activation of MOR with endomorphin-2 also increased the diffusion coefficient of TRPV1 (D = 1.150 ± 0.121 µm^2^/s), but less than capsaicin (Figure 4A). Importantly, pretreatment of the cells with naloxone or capsazepine prevented the agonist-induced changes in the receptor diffusion.

Treatment of HMY-1/TRPV1 cells with both capsaicin and endomorphin-2 changed the proportion of mobile and immobile TRPV1 in the plasma membrane. Whereas capsaicin reduced the mobile fraction of TRPV1, endomorphin-2 markedly increased the mobile fraction of TRPV1 (Figure 4B). Pretreatment of the cells with naloxone or capsazepine before adding endomorphin-2 or capsaicin prevented the effects of both these agonists.

### 2.4. Knockdown of β-Arrestin 2 Prevents Activation-Induced Changes in the Mobility of Both TRPV1 and MOR

In order to explore the possible role of β-arrestin 2 in modulating the mobility of MOR and TRPV1 in the plasma membrane, the receptor diffusion was monitored in HMY-1/TRPV1 cells after knockdown of β-arrestin 2. The efficacy of siRNA-mediated β-arrestin 2 knockdown was confirmed by Western blotting. This analysis indicated that the expression of β-arrestin 2 was downregulated by about 90% in cells transfected with β-arrestin 2 siRNA. Interestingly, knockdown of β-arrestin strongly affected receptor diffusibility and limited the modulatory effects of agonists on receptor movement.

**Figure 5:**
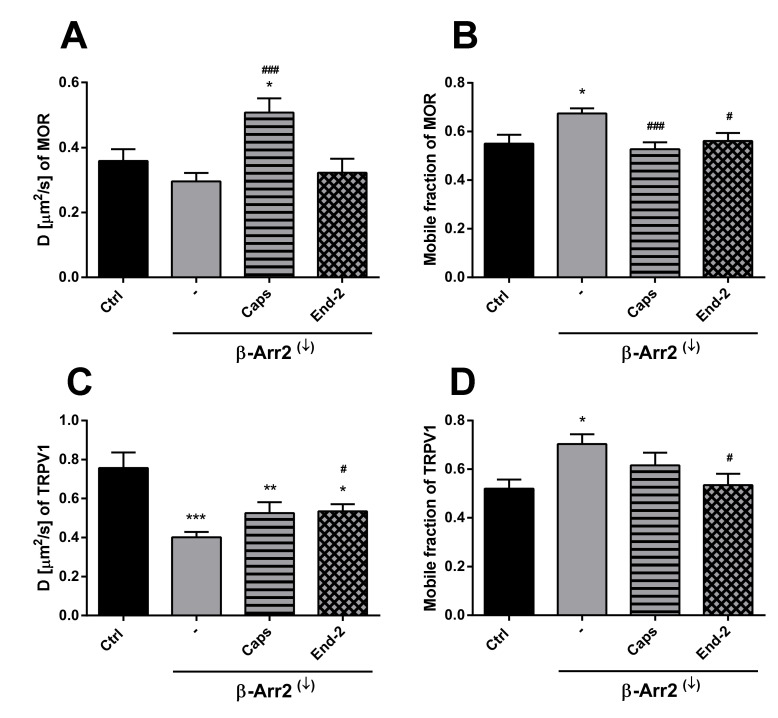
Effect of β-arrestin 2 knockdown on the ability of capsaicin and endomorphin-2 to affect the lateral mobility of MOR and TRPV1 in the plasma membrane of HMY-1/TRPV1 cells. The diffusion coefficients (**A**,**C**) and the mobile fractions (**B**,**D**) of TRPV1 (upper panel) and MOR (lower panel) were obtained from FRAP measurements. The knockdown of β-arrestin 2 (β-Arr2^(↓)^) was performed 24 h before addition of the ligands. The cells plated in a glass bottom chamber were treated with capsaicin (Caps, 0.5 µM) or endomorphin-2 (End-2, 1 µM) for 5 min before measurements. FRAP experiments were performed on the bottom cell membrane using a Zeiss LSM 880 confocal microscope. The data were collected from three independent experiments, at least 50 cells in each group. Results are expressed as means ± S.E.M. Asterisks or hashes denote significant differences between control (Ctrl) or untreated (-) β-Arr2^(↓)^ cells and different drug treatment groups (* *p* < 0.05, ** *p* < 0.01, *** *p* < 0.001 compared to corresponding control; ^#^ *p* < 0.05, ^###^ *p* < 0.001 compared to corresponding β-Arr2^(↓)^ (-)).

In the case of MOR, knockdown of β-arrestin 2 somewhat decreased (by about 16%) the lateral mobility of unactivated MOR (Figure 5A). Moreover, this intervention markedly attenuated the ability of capsaicin and endomorphin-2 to affect the rate of diffusion of MOR. Whereas capsaicin increased the rate of MOR diffusion by 42%, endomorphin-2 did not change the receptor movement under these conditions. Interestingly, the proportion of mobile MOR significantly increased (by about 18%) after β-arrestin 2 knockdown (Figure 5B). Capsaicin reduced the MOR mobile fraction (by 22%) in HMY-1/TRPV1 cells with suppressed expression of β-arrestin 2 to a similar extent as in the cells carrying a normal level of β-arrestin 2 (decrease by about 21%). Addition of endomorphin-2 to HMY-1/TRPV1 cells after β-arrestin 2 knockdown reduced the MOR mobile fraction by about 17%. β-Arrestin 2 knockdown substantially decreased (by about 47%) the lateral mobility of TRPV1 and its rate of diffusion was increased to the same extent (by about 25%) by the addition of capsaicin or endomorphin-2 (Figure 5C). The fraction of mobile receptors was significantly increased after knockdown of β-arrestin 2 (by about 25%) and the addition of capsaicin or endomorphin-2 reduced the proportion of mobile TRPV1 back to the control level (Figure 5D). This was in contrast to normal MY-1/TRPV1 cells, where endomorphin-2 significantly increased (by about 20–30%) the proportion of mobile MOR and TRPV1.

### 2.5. Functional Studies of MOR and TRPV1 Signaling

**Figure 6:**
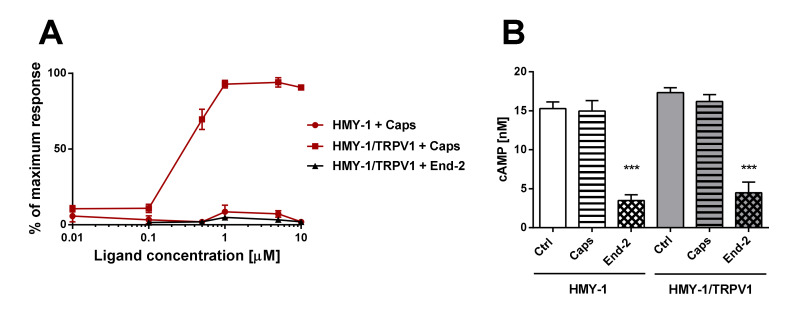
Functional studies of TRPV1- and MOR-mediated signaling. (**A**) The effect of increasing concentrations of capsaicin (Caps) and endomorphin-2 (End-2) on intracellular levels of calcium in HMY-1 and HMY-1/TRPV1 cells was determined using endpoint calcium assay described in Section 4. The graph shows calcium response to agonist stimulation expressed as percentage of the maximum response. (**B**) The effect of capsaicin and endomorphin-2 (both 1 µM) on cAMP production in HMY-1 and HMY-1/TRPV1 cells incubated in the presence of forskolin was determined using TRF assay described in Section 4. Results are expressed as means ± S.E.M. (*** *p* ≤ 0.001 compared to corresponding control).

In this set of experiments, we examined agonist-induced Ca^2+^ responses and inhibition of adenylyl cyclase (AC) activity in HMY-1 and HMY-1/TRPV1 cells. We found that the TRPV1 agonist capsaicin was able to evoke a dose-dependent Ca^2+^ influx only in HMY-1/TRPV1 cells (Figure 6A). No such response was observed on HMY-1 cells. The MOR agonist endomorphine-2 did not elicit any detectable Ca^2+^ responses at any concentration. The ability of receptor agonists to affect forskolin-stimulated AC was determined using the HTRF assay (Cisbio Bioassays). Whereas capsaicin did not change AC activity either in HMY-1 or HMY-1/TRPV1, endomorphin-2 inhibited the enzyme activity in both these cell lines to a similar extent (Figure 6B).

### 2.6. Activation of TRPV1 and MOR Alters the Plasma Membrane Localization of β-Arrestin 2

**Figure 7:**
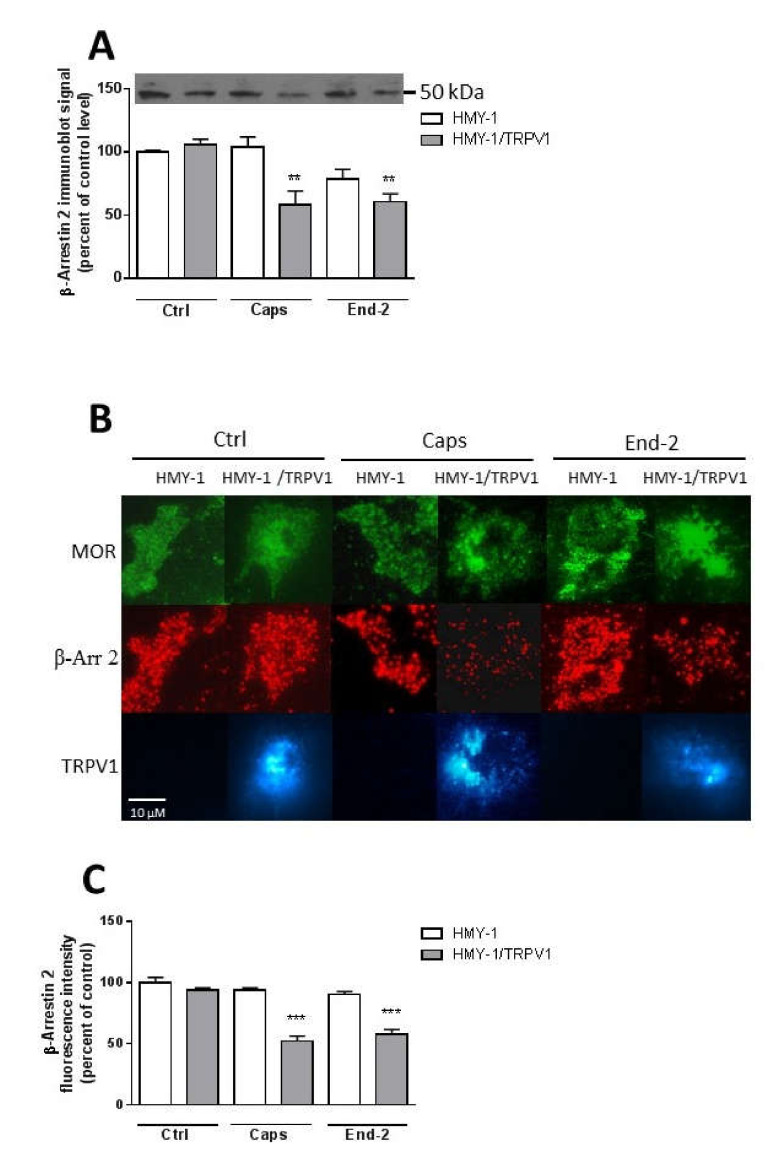
Effect of capsaicin and endomorphin-2 on the association of β-arrestin 2 with the plasma membrane. (**A**) Immunoblot analysis of β-arrestin 2 distribution in the plasma membrane fractions isolated from HMY-1 and HMY-1/TRPV1 cells that were incubated for 5 min in the absence (Ctrl) and presence of 1 µM capsaicin (Caps) or endomorphin-2 (End-2). The figure shows a representative Western blot from four independent experiments. The band intensities were densitometrically evaluated and normalized to β-actin levels. Results represent means ± S.E.M. and are expressed as percentage of control (** *p* < 0.01 versus control). (**B**) Immunofluorescence detection of β-arrestin 2, TRPV1 and MOR in HMY-1 and HMY-1/TRPV1 cells that were incubated for 5 min in the absence (Ctrl) and presence of 1 µM capsaicin (Caps) or endomorphin-2 (End-2). After treatment with the agonists, the cells were fixed with 4% PFA and immunostained using primary anti-β-arrestin 2 antibody and secondary Alexa Fluor 594-conjugated antibody. Cells were visualized using Zeiss Elyra SP.1 nanoscope equipped with TIRF technology. (**C**) Relative intensity of fluorescence signals corresponding to the amount of β-arrestin 2 associated with the plasma membrane were quantified by Image J software. Results represent means ± S.E.M. and are expressed as percentage of control (*** *p* < 0.001 compared to corresponding control).

We were interested to determine whether stimulation of HMY-1 and HMY-1/TRPV1 affected the association of β-arrestin 2 with the plasma membrane. After incubating the cells for 5 min in the presence of capsaicin or endomorphin-2, the relative level of β-arrestin 2 in the plasma membrane fraction was assessed by immunoblot analysis. Both capsaicin and endomorphin-2 markedly reduced (by about 50%) the content of β-arrestin 2 in samples of plasma membranes from HMY-1/TRPV1 cells (Figure 7A). There was no such change in the plasma membrane distribution of β-arrestin 2 after treatment of HMY-1 cells by these agonists.

Then, we investigated the localization of β-arrestin 2 at the plasma membrane using TIRF microscopy. HMY-1 and HMY-1/TRPV1 cells stained with antibody against β-arrestin 2 and Alexa Fluor 594-conjugated secondary antibody were visualized with Zeiss Elyra SP.1 nanoscope (Figure 7B). These experiments indicated that transfection of HMY-1 cells with TRPV1 did not change the level of β-arrestin 2 associated with the plasma membrane (Figure 7C). Interestingly, pretreatment of HMY-1/TRPV1 cells, but not HMY-1 cells, with capsaicin and endomorphin-2 reduced the plasma membrane localization of β-arrestin 2.

### 2.7. Crosstalk between TRPV1 and MOR Signaling Is Driven via the MAPK ERK1/2 Pathway

**Figure 8:**
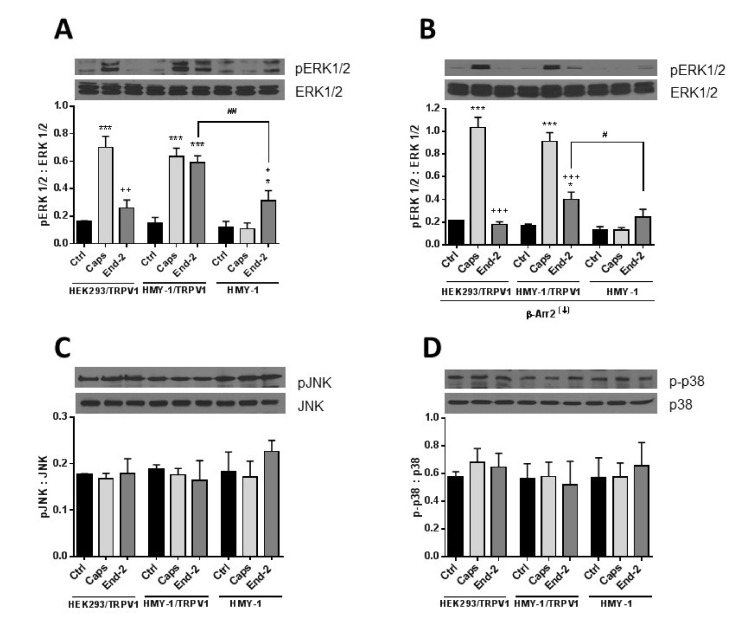
Effect of capsaicine and endomorphin-2 on phosphorylation of MAP kinases. HEK293/TRPV1, HMY-1/TRPV1, and HMY-1 cells were incubated for 5 min in the absence (Ctrl) and presence of 1 µM capsaicin (Caps) or endomorphin-2 (End-2). In some experiments, β-arrestin 2 was knocked down (β-Arr2^(↓)^) before treatment of the cells with the agonists. Aliquots of whole cell lysates (30 µg protein/lane) were resolved by SDS-PAGE and subjected to immunoblotting using specific antibodies against ERK1/2 and pERK1/2 (**A**,**B**), p38 and p-p38 (**C**), and JNK and pJNK (**D**). Shown are representative blots from four independent experiments. The band intensities were densitometrically evaluated and normalized to β-actin levels. Results are expressed as means ± S.E.M. of the ratios of phosphorylated to unphosphorylated MAPK forms (* *p* < 0.05, *** *p* < 0.001 compared to corresponding control; ^#^ *p* < 0.05, ^##^ *p* < 0.01 compared to HMY-1/TRPV1; ^+^ *p* < 0.05, ^++^ *p* < 0.01, ^+++^ *p* < 0.001 compared to cells treated with Caps).

It seems obvious that β-arrestin 2 can play an important role in mediating crosstalk between TRPV1 and MOR. To decipher the potential involvement of post-receptor signaling pathways in cross- communication between both these receptors, we monitored protein phosphorylation changes of key components of the MAPK signaling cascades in response to stimulation of HEK293/TRPV1, HMY-1/TRPV1, and HMY-1 cells by capsaicin and endomorphin-2. Phospho- and total protein levels of ERK1/2, p38 and JNK were assessed by immunoblotting (Figure 8). Capsaicin remarkably increased phosphorylation of ERK1/2 in cells expressing TRPV1 (HEK293/TRPV1 and HMY-1/TRPV1). No significant change occurred in HMY-1 cells. Endomorphin-2 increased phosphorylation of ERK1/2 in cells expressing MOR (HMY-1 and HMY-1/TRPV1). Intriguingly, a much greater elevation of ERK1/2 phosphorylation was found in cells expressing both MOR and TRPV1 (Figure 8A). We performed yet another set of experiments on cells with reduced expression of β-arrestin 2. Knockdown of β-arrestin 2 did not affect capsaicin-induced phosphorylation of ERK1/2, but strongly attenuated the ability of endomorphin-2 to increase phosphorylation of ERK1/2 in the cells expressing both MOR and TRPV1 (Figure 8B). The phosphorylation levels of the other two MAP kinases, p38 and JNK, were not changed in either cell line after treatment with both agonists (Figure 8C,D).

## 3. Discussion

The purpose of this study was to investigate the relationship between MOR and TRPV1 and potential crosstalk between their signaling pathways. Using Fluorescent recovery after photobleaching (FRAP), we compared the mobility of both these receptors in the plasma membrane after their activation by capsaicin and endomorphin-2. Capsaicin is a naturally occurring vanilloid that exhibits TRPV1 agonistic activity [15]. Endomorphin-2 is an endogenous MOR agonist biased towards β-arrestin 2-dependent signaling [16,17]. We have previously found that the lateral mobility of MOR in the plasma membrane was significantly increased by DAMGO and decreased by endomorphin-2 [14]. Our current results indicate that the exogenous expression of TRPV1 in HMY-1 cells did not affect the mobility of MOR. However, the activation of TRPV1 with capsaicin markedly increased the mobility of MOR and decreased the number of mobile receptors in the plasma membrane. On the other hand, we have also observed that the diffusion rate of TRPV1 was changed not only after activation of TRPV1 with capsaicin but also after activation of MOR with endomorphin-2. Interestingly, there were similar changes in mobile fractions of both MOR and TRPV1 after activation with endomorphin-2 or capsaicin. The mobile fractions of both these receptors were decreased after treatment with capsaicin and increased after treatment with endomorphin-2. To date, there is not much information about TRPV1 diffusion in the plasma membrane. It has been reported that the mobility of TRPV1 decreased within seconds upon channel activation in the presence of Ca^2+^ [18]. We therefore presume that the decrease in the mobile fraction observed after activation of TRPV1 with capsaicin is due to the certain number of active channels in the plasma membrane.

Our functional studies confirmed that the activation of TRPV1 by capsaicin leads to calcium influx and the activation of MOR by endomorphin-2 suppresses AC activity as reflected by a decrease in cAMP production. Although capsaicin did not affect AC activity and endomorphin-2 did not trigger calcium influx, both these ligands were able to change the mobility of their cognate as well as noncognate receptors in the plasma membrane. These data suggest that these receptors may communicate with each other, but the mechanism is not yet known.

β-Arrestins are important proteins that can form scaffolding complexes with a wide variety of proteins in order to regulate diverse signaling pathways [19]. β-Arrestin 2 was first identified as a protein capable of mediating desensitization of β_2_-adrenergic receptor signaling after agonist stimulation [20]. It is nowadays obvious that β-arrestins play an important role in cell signaling including GPCR desensitization and, rather curiously, may also participate in desensitization of TRPV1 [21]. It was recently shown that the activation of TRPV1 results in nuclear translocation of GRK5, which blocks its ability to phosphorylate MOR, and that this interaction leaves the G protein-mediated analgesic signaling of MOR intact but inhibits β-arrestin 2-mediated internalization and desensitization of MOR [22]. These authors hypothesized that MOR and TRPV1 may compete for GRK5 and β-arrestin 2. Our current observations indicate that β-arrestin 2 is crucially implicated in mediating the relationship between MOR and TRPV1. According to the classical scenario, activation of MOR with endomorphin-2 initiates GRK-catalyzed receptor phosphorylation, which is followed by β-arrestin 2 recruitment to the plasma membrane and binding to the receptor. Consequently, the amount of β-arrestin 2 in the cytoplasm is lowered, which may rather limit its scaffolding function. This could likely explain the observed changes in the diffusion coefficient and mobile fraction of TRPV1.

In order to assess the role of β-arrestin 2 in TRPV1 and MOR cross-communication, we investigated receptors mobility after β-arrestin 2 knockdown. As expected, this intervention increased the mobile fraction of both TRPV1 and MOR in plasma membranes of control (unstimulated) cells. In parallel, the diffusion coefficient of TRPV1 was markedly reduced and there was a downward tendency in the diffusion rate of MOR. The effects of capsaicin and endomorphin-2 on the mobility of both TRPV1 and MOR were notably diminished or altered after β-arrestin 2 knockdown. Interestingly, the study of Por and colleagues [23] demonstrated increased association of unactivated TRPV1 and β-arrestin 2 in CHO cells under normal serum media conditions and reduced association of these two proteins in serum-free media. Here, we observed that treatment of HMY-1/TRPV1 cells with capsaicin for 5 min resulted in a significant decrease in the amount of β-arrestin 2 attached to the plasma membrane. It has been demonstrated that the activation of MOR by some ligands including endomorphin-2 leads to internalization of the MOR–β-arrestin 2 complex [17]. Although this event is quite fast, it does not occur within 5 min after MOR activation [24]. It therefore seems plausible that the observed decrease in plasma membrane β-arrestin 2 level is somehow connected with TRPV1 signaling. Our data supports this view: the activation of MOR with endomorfin-2 in HMY-1 cells (not expressing TRPV1) did not reduce the amount of β-arrestin 2 in the plasma membrane. Surprisingly, stimulation of HMY-1/TRPV1 cells with endomorfin-2, similarly as with capsaicin, led to a significant decrement in β-arrestin 2 in the plasma membrane. These observations endorse our hypothesis that there is crosstalk between MOR- and TRPV1-mediated pathways which can play an important role in modulating the receptor signaling.

The MAPK family is a diverse group of proteins involved in ensuring a wide variety of cell physiological processes. They include extracellular signal-regulated kinase (ERK), p38-mitogen activated protein kinase (p38), and c-Jun N-terminal kinase (JNK) [25]. It has been found that the activation of MAPK may participate in generating pain hypersensitivity and that MEK inhibitors known to suppress phosphorylation of ERK can effectively alleviate pain at various time points in several animal models of neuropathic pain [26,27]. It has been reported that stimulation of TRPV1 with capsaicin leads to a rapid phosphorylation and activation of ERK1/2 [28]. Morphine was found to induce ERK activation in CHO cells stably transfected with MOR [29]. It seems evident that MOR can mediate responses in an agonist dependent manner. Agonists such as morphine and methadone activate ERKs via the protein kinase C-dependent pathway but not the β-arrestin-dependent pathway. Contrarily, agonists such as etorphine and fentanyl activate ERKs in a β-arrestin-dependent manner [30]. Previous studies have indicated that morphine and methadone are G protein biased agonists, whereas etorphine, fentanyl, and endomorphin-2 are β-arrestin biased agonists [17]. Interestingly, β-arrestin 2 is not a regulator of phosphorylation of ERK1/2 initiated by activation of some GPCRs [31,32]. Here we show that the cross-communication between the TRPV1 and MOR signaling pathways after activation either by capsaicin or by endomorphin-2 proceeds to ERK1/2. This is clearly evident from our results showing the increase in ERK1/2 phosphorylation after activation of TRPV1 with capsaicin in cells expressing TRPV1. Endomorphin-2 elicited a significant increase in ERK1/2 phosphorylation in cells expressing MOR but, rather surprisingly, a more pronounced increase in ERK1/2 phosphorylation was detected in cells expressing both MOR and TRPV1. Interestingly, Popiolek-Barczyk et al. [33] reported that inhibition of ERK1/2 phosphorylation through inhibiting MEK1/2 (kinase phosphorylating ERK1/2) significantly enhanced the analgesic effects of morphine. It means that phosphorylation of ERK1/2 attenuates analgesia induced by opioids. On the other hand, the p38 MAPK pathway was found to be responsible for more effective analgesia after morphine administration [34]. Here, we observed that TRPV1, which plays a central role in nociception, is of high importance for enhanced phosphorylation of ERK1/2 but not for p38 or JNK phosphorylation in HMY-1/TRPV1 cells following activation of MOR with endomorphin-2. Interestingly, β-arrestin 2 appears to be especially important for MOR-induced ERK1/2 phosphorylation because its downregulation strongly attenuated the ability of endomorphin-2 to increase ERK1/2 phosphorylation. On the other hand, the ability of capsaicin to increase ERK1/2 phosphorylation was not affected by β-arrestin 2 knockdown. These observations suggest that the mechanism of receptor-induced ERK1/2 activation somewhat differ between TRPV1 and MOR.

## 4. Materials and Methods

### 4.1. Cell Culture, Transient Transfection, and Drug Treatment

The human embryonic kidney (HEK) 293 cell line was purchased from Sigma-Aldrich (St. Louis, MI, USA). The HMY-1 cell line (HEK293 cells stably expressing MOR) was prepared and characterized previously [14]. Both cell lines were cultured in Dulbeco’s modified Eagle’s medium (DMEM) supplemented with 10% fetal bovine serum (FBS) and 1% antibiotic antimycotic solution (AAS, Sigma-Aldrich) at 37 °C in a humidified atmosphere containing 5% CO_2_. For transient transfection, cells were plated in the appropriate multi-well plates and cultivated in the above-mentioned medium. After 24 h, on reaching 60–70% confluence, cells were transfected using Lipofectamine 3000 reagent (Invitrogene, Waltham, MA, USA) according the manufacturer’s instructions. Briefly, Lipofectamine 3000 diluted in Opti-MEM medium was mixed with DNA or siRNA diluted in Opti-MEM with P3000 reagent. Final mixture was incubated for 5 min at room temperature and then added directly to the cell culture. After 24 h of incubation, cells were used in further experiments. The plasmid containing TRPV1 with CFP fused to the C-terminus was a generous gift from Dr. Leon D Islas (National Autonomous University of Mexico [35]. β-Arrestin 2 siRNA (sc-29208) was purchased from Santa Cruz Biotechnology (Dallas, TX, USA). HMY-1 cells carrying the TRPV1–CFP fusion receptor were denoted HMY-1/TRPV1.

The function of MOR and TRPV1 was modulated by the MOR agonist endomorphin-2 or antagonist naloxone and the TRPV1 agonist capsaicin or antagonist capsazepine. These ligands were dissolved in phosphate-buffered saline (PBS). Before starting the measurement, cells were incubated for 5 min either with endomorphin-2 (1 µM) or capsaicin (0.5 µM) or naloxone (10 µM) or capsazepine (5 µM). In some cases, cells were pretreated for 10 min with naloxone (10 µM) or capsazepine (5 µM) and then exposed for 5 min to endomorphin-2 (1 µM) or capsaicin (0.5 µM), respectively.

### 4.2. Total Internal Reflection (TIRF)

Cells were plated on a 35 mm glass-bottom dishes in phenol red-free DMEM supplemented with 10% FBS and 1% AAS. After 24 h, cells were transfected with plasmid cDNA encoding the CFP-tagged TRPV1 receptor as described above. One day after transfection, cells were visualized using Zeiss Elyra SP.1 nanoscope equipped with TIRF technology.

### 4.3. Fluorescent Recovery after Photobleaching

HMY-1/TRPV1 cells were seeded on glass-bottom dishes and maintained in phenol red-free DMEM supplemented with 10% FCS, 1% AAS, and 0.8 mg/mL geneticin. FRAP experiments on living cells were performed on an inverted Zeiss LSM 880 confocal laser scanning microscope (Carl Zeiss AG, Oberkochen, Germany) equipped with 40×/1.2 WDICIII C Apochromat objective lens and back-thinned CCD camera (Zeiss Axio Cam). For excitation of fluorophores, we used the 514-nm laser for visualizing YFP and 405-nm laser for visualizing CFP. Images were acquired using ZEN Black software (Carl Zeiss AG). During all FRAP experiments, the cells were placed in a chamber providing a stable temperature 37 °C and 5% CO_2_. All the diffusion data was always assessed by FRAP measurements in plasma membrane areas adjacent to the glass support. Bleaching (six iterations per bleach) was accomplished with a circular spot with 2-μm radius using the 488- and 514-nm for YFP and 458- and 488-nm for CFP laser pulse from a 40-mW Argon laser operating at 100% power. The time course of the fluorescence recovery signal after photobleaching was monitored at low laser intensity (2% of maximum power) and 512 × 512 pixels resolution with sampling rate of 2 ms. In each FRAP series, 15 prebleach images were collected and, immediately after photobleaching, 400 successive postbleach images were recorded to monitor the fluorescence redistribution. FRAP curves were normalized to the prebleach value of the respective pulse train. The data obtained from at least 50 cells (screened in three individual runs) during each FRAP experiment were analyzed using easyFRAP, a MATLAB platform-based tool [36]. Recovery curves were calculated according to the following equation:

recovery (%) = (I_bleach_ − I_bckg_)/(I_ref_ − I_bckg_) × 100

where I_bleach_ is the fluorescence intensity of the bleached spot, I_bckg_ is the fluorescence intensity of the background, and I_ref_ is the fluorescence intensity of the control regions in other cells or regions far remote from the target cell. The values of the apparent diffusion coefficients (D) for both receptors were obtained from the following equation:

D = 0.224 ω^2^/t_1/2_

where ω is the diameter of the selected bleached spot and t½ is the half-life of the fluorescence recovery [37].

The potential effects of agonists on the lateral mobility of MOR–YFP or TRPV1–CFP were investigated using endomorphin-2 and capsaicin. HMY1/TRPV1 cells were pretreated for 5 min with individual agonists and the agonists were left in the medium during the whole experiment. Antagonists of MOR and TRPV1 receptors (naloxone and capsazepine, respectively) were applied to cells 10 min before adding agonists and they were used at final concentrations 10 times higher than those of agonists.

### 4.4. Time Resolved Fluorescence Assay for cAMP

The direct quantitative determination of cAMP in cells affected by receptor ligands was performed using cAMP–Gi kit (Cisbio Bioassays, Parc Marcel Boiteux, Codolet, France) based on HTRF^®^ technology (Homogenous Time Resolved Fluorescence). Cells were seeded in a 384-well plate (8000 cells per well), transfected in 24 h, and the assay was performed after 24 h of incubating the cells with complex of Lipofectamine 3000-TRPV1 DNA plasmid. Cells were incubated for 20 min at 37 °C and 5% CO_2_ in the presence of ligands diluted to a final concentration 1 µM in stimulation buffer. Forskolin at a concentration 2 µM was added before incubating the cells for 45 min at 37 °C. Then cryptate-labeled cAMP and monoclonal anti-cAMP-d2 antibody diluted in lysis and detection buffer were added. The plate was incubated for 1 h at room temperature before transferring the final lysate to a white 96-well plate and reading absorbance at 620 and 665 nm in Tecan Safire 2 reader.

### 4.5. Endpoint Calcium Assay

Cells were seeded in a 384-well plate (8000 cells per well) and after 24 h transfected with the TRPV1–CFP plasmid. After the next 24 h, cells were used for the assay. We used Cell MeterTM No Wash and Probenecid-Free Endpoint Calcium Assay kit (AAT Bioquest). Fluo-8ETM AM dye solution was prepared according to the manufacturer’s instructions and mixed with assay buffer. Then, 25 µL per well of the mixture was added to the plate and incubated for 45 min at 37 °C. After the incubation, agonist resuspended in HHBS buffer was added and the calcium flux assay was run immediately. The fluorescence intensity at Ex/Em = 490/525 nm was monitored on a microplate reader (bottom read mode).

### 4.6. Isolation of a Plasma Membrane Fraction

Transfected and non-transfected (control) cells were incubated in the presence or absence of ligands at 37 °C for 5 min. The cells were immediately chilled in an ice bath and harvested in TMES buffer (20 mM Tris, 3 mM MgCl2, 1 mM EDTA, 250 mM sucrose; pH 7.4). The plasma membrane fraction was isolated using Percoll^®^ self-forming gradient as previously described with some modifications [38]. Cell homogenates (3 mL) were loaded on the top of 23 mL of 18% Percoll solution in TMES buffer and centrifuged in a Beckman Ti50 rotor at 60,000× g for 15 min. The resulting upper layer enriched in plasma membranes was collected, diluted in TME buffer (20 mM Tris, 3 mM MgCl_2_, 1 mM EDTA; pH 7.4), and centrifuged at 150,000× *g* for 1 h. The pellet was resuspended in TME buffer, frozen in liquid nitrogen, and stored at −80 °C.

### 4.7. SDS-PAGE and Immunoblotting

Samples were solubilized in Laemmli buffer and loaded on standard 10% acrylamide gels for SDS-PAGE. The resolved proteins were transferred to nitrocellulose membrane, blocked with 5% non-fat dry milk in TBS buffer (10 mM Tris, 150 mM NaCl; pH 8.0) for 30 min, and then incubated in the presence of relevant primary antibodies with gentle agitation overnight at 4 °C. After three 10-min washes in TBS containing 0.3% Tween 20 (TBS-Tween), the secondary antibodies labeled with horseradish peroxidase were applied for 1 h at room temperature. After another three 10-min washes in TBS-Tween, the blots were visualized by enhanced chemiluminescence technique according to the manufacturer’s instructions (Pierce Biotechnology, Rockford, IL, USA). The immunoblots were scanned and quantitatively analyzed by ImageJ software.

### 4.8. Immunofluorescence

Cells were plated in 35 mm glass bottom dishes and were cultured in phenol red-free DMEM supplemented with 10% FBS and 1% AAS for 24 h prior to transfection with TRPV1-CFP construct as described above. The following day, cells were fixed with 4% paraformaldehyde for 15 min at room temperature. Cells were washed three times in PBS and permeabilized with 0.2% Triton in PBS for 5 min. Samples were blocked with 10% donkey serum and 1% BSA in PBS for 1 h and then washed three times and incubated with primary antibody against β-arrestin 2 for 1 h. Cells were washed three times with PBS and incubated for 1 h with secondary donkey anti-rabbit antibody (Alexa Fluor 594, Life Technologies). After incubation, cells were washed three times with PBS and visualized using Zeiss Elyra SP.1 nanoscope enabling live-cell imaging and TIRF illumination.

### 4.9. Materials

Lipofectamine 3000 was purchased from Invitrogen (Carlsbad, CA, USA) and fetal bovine serum (FBS) was from Thermo Fisher Scientific (Waltham, MA, USA). HTRF cAMP Gi Assay kit was purchased from Cisbio Bioassays (Barford, MA, USA) and Cell MeterTM No Wash and Probenecid-Free Endpoint Calcium Assay kit was from AAT Bioquest (Sunnyvale, CA, USA). β-Arrestin 2 antibody was from ThermoFisher Scientific, ERK1/2, p-ERK1/2, JNK, and pJNK antibodies were from Cell Signaling Technology (Beverly, MA) and p38 and p-p38 antibodies were from Santa Cruz Biotechnology. All the ligands and other chemicals were purchased from Sigma-Aldrich (St. Louis, MI, USA) and they were of the highest purity available.

### 4.10. Statistics

Data are expressed as mean values ± standard error of the mean (S.E.M.) of at least three independent experiments. All statistical analyses were conducted using GraphPad Prism, Version 6.0 (GraphPad Software Inc., La Jolla, CA, USA). The differences between the means of relevant groups were statistically evaluated by one-way ANOVA followed by the Bonferroni post hoc test. Significance level was set at *p* ≤ 0.05.

## 5. Conclusions

Taken together, our results indicate that not only the activation of one receptor influences the other one but that the mere presence of one receptor can modulate the signaling properties of the other receptor. To date, there is no information about the possible formation of dimers between TRPV1 and MOR in the plasma membrane. The different lateral mobility properties of both these receptors insinuate that this option is rather unlikely. Nevertheless, the present data clearly demonstrate that capsaicin and endomorphin-2 may both influence the behavior of TRPV1 and MOR in the plasma membrane and shed some new light on the possible cross-communication between these two receptors and their signaling pathways.
